# A Novel Feature Optimization for Wearable Human-Computer Interfaces Using Surface Electromyography Sensors

**DOI:** 10.3390/s18030869

**Published:** 2018-03-15

**Authors:** Han Sun, Xiong Zhang, Yacong Zhao, Yu Zhang, Xuefei Zhong, Zhaowen Fan

**Affiliations:** Department of Electronic Science and Engineering, Southeast University, Nanjing 210096, China; 230139593@seu.edu.cn (H.S.); 220161239@seu.edu.cn (Ya.Z.); 230129600@seu.edu.cn (Yu.Z.); zxf@seu.edu.cn (Xu.Z.); zhaowenfan@seu.edu.cn (Z.F.)

**Keywords:** human-computer interface, surface electromyogram, channel selection, feature optimization, multi-class recognition, support vector machine

## Abstract

The novel human-computer interface (HCI) using bioelectrical signals as input is a valuable tool to improve the lives of people with disabilities. In this paper, surface electromyography (sEMG) signals induced by four classes of wrist movements were acquired from four sites on the lower arm with our designed system. Forty-two features were extracted from the time, frequency and time-frequency domains. Optimal channels were determined from single-channel classification performance rank. The optimal-feature selection was according to a modified entropy criteria (EC) and Fisher discrimination (FD) criteria. The feature selection results were evaluated by four different classifiers, and compared with other conventional feature subsets. In online tests, the wearable system acquired real-time sEMG signals. The selected features and trained classifier model were used to control a telecar through four different paradigms in a designed environment with simple obstacles. Performance was evaluated based on travel time (TT) and recognition rate (RR). The results of hardware evaluation verified the feasibility of our acquisition systems, and ensured signal quality. Single-channel analysis results indicated that the channel located on the extensor carpi ulnaris (ECU) performed best with mean classification accuracy of 97.45% for all movement’s pairs. Channels placed on ECU and the extensor carpi radialis (ECR) were selected according to the accuracy rank. Experimental results showed that the proposed FD method was better than other feature selection methods and single-type features. The combination of FD and random forest (RF) performed best in offline analysis, with 96.77% multi-class RR. Online results illustrated that the state-machine paradigm with a 125 ms window had the highest maneuverability and was closest to real-life control. Subjects could accomplish online sessions by three sEMG-based paradigms, with average times of 46.02, 49.06 and 48.08 s, respectively. These experiments validate the feasibility of proposed real-time wearable HCI system and algorithms, providing a potential assistive device interface for persons with disabilities.

## 1. Introduction

Human-computer interfaces (HCI) for those with motor deficits based on bioelectrical signals have received increasing attention in the last decade. HCI provides communication and control channels between human subjects and the surrounding environment with the purpose of replacement or augmentation of muscle activity [1]. Common classes of bio-signals used to control assistive devices include electromyography (EMG) [2,3], electroencephalography (EEG) [4,5], electrooculography (EOG) [6,7], and fusions of these signals [8,9,10,11]. 

This work presents the performance results of an HCI system based on electrical currents generated in the contraction and relaxation phase of muscles [12] known as sEMG. Measurement of sEMG is noninvasive, can offer excellent signal-to-noise ratio compared to EEG, and provides real-time information about muscle intents [13]. 

Applications of sEMG include early disease detection [14], seizure [15,16] and fall detection [10,17], gesture [18,19], and sign language recognition [3,20]. These applications recorded activities of facial [21,22], upper [18,23] and lower-limb [17,24] muscles.

The sEMG-based HCIs are sensitive to electronic noise, movement artefacts and muscle fatigue [12]. To overcome the problem, wearable rigid printed circuit board (PCB)-based systems are often used [3,25,26]. However, these devices still need to be fully fixed and tethered. Another solution is development of wearable and flexible electronics. These sEMG sensors have been utilized to monitor electrocardiogram (ECG), EMG and body posture [27,28] signals as well as to detect arm gestures [29].

The sEMG-based HCIs require effective features to provide real-time efficiency from the data processing standpoint. Time-domain feature extraction is the most common method because these features provide high recognition rate and low computational cost. Root mean square (RMS) [30,31,32,33] and mean absolute value (MAV) [29,33,34] are most popular among these features. Time-frequency domain features which characterize varying frequency information at different time locations have received attention recently. Time-frequency domain analysis can efficiently eliminate the non-stationary noise in either time or frequency domain [12,35,36,37,38]. 

Feature optimization/selection techniques further enhance the performance. These techniques include filter, wrapper, and embedded methods [39]. Filter and wrapper methods have very low computational costs because they optimize features independent of the classification performance [40,41]. In contrast, embedded methods rely on criteria that are generated during the classifier training process. Examples of embedded techniques are the support vector machine (SVM)-based Recursive Feature Elimination (RFE) [42] and the linear discriminant analysis (LDA)-based Fisher’s Discriminant (FD) function [17]. 

Channel selection is another simple feature selection/optimization technique. Within this method, channel optimization is based on the single-channel classification accuracy. A more sophisticated adaptation is about selecting important muscles/channels that highly contribute to the specific movements according to the power and frequency distribution of sEMG signals [31].

Classification is the next step to distinguish between different movements. Simple and linear classifiers are preferable because of their simplicity and ease of implementation. For example, Linear Discriminant Analysis (LDA) [29], k-Nearest Neighbor (kNN) [3] and Decision Tree (DT) [3,13] are feasible to separate small numbers of uncomplicated classes clearly. To increase expandability and performance in complicated systems, Support Vector Machine (SVM) [43], Artificial Neural Network (ANN) [44], Fuzzy Min-Max Neural Network (FMMNN) [40] and Random Forest (RF) [37] are suggested for classification. Neural Network classifiers can be used for both simple and complex cases due to their high performance. However, with too many hidden layers or hidden units, the classifiers need long training times and require large amounts of training data. Unsupervised learning methods including K-Means and Fuzzy C-Means have also provided high classification performance in sEMG recognition [32].

An important application of biopotential-based HCI is smart wheelchair control. The conventional paradigm of smart wheelchair control is through joysticks [45]. This paradigm is not applicable for patients with low or no control of their upper limb. The sEMG-based HCI is an advanced alternative controller [46,47], however with more sophisticated implementation than the conventional joystick control. A critical problem is the functionality requirement for accurate control. (1) The recognition for more motions or gestures can increase degree-of-freedom [37,42]. (2) A state-machine based control and a proportional control could meet the requirement [48,49]. In this method, a modality or a channel is used to switch control modes. (3) Fusion of two or more types of sensors can realize the high-dimensional control for assistive devices. The combination of sEMG and inertial measurement units (IMU) consisted of conventional accelerometers (ACC) and gyroscopes is frequently used because of the high performance in long-term control. Muscle fatigue and skin sweat can make sEMG signals drift, while they have no influence to IMU signals [50,51].

In this paper, we explore the performance of sEMG-based HCI in controlling a telecar. We present a wireless wearable sEMG system based on flexible printed circuit (FPC) with embedded dry metallic detecting electrodes that avoids channels connection and fixation challenges. We also present methodology to select sEMG feature subsets for the recognition of four movements. The novel entropy criteria (EC) and Fisher discrimination (FD) criteria are compared with the conventional RFE method. Dunn-Heriksen et al. [52] introduced EC in EEG channel selection. Here we adopt EC for sEMG feature optimization. Fisher’s discriminant based separability measurement has been widely utilized in feature optimization [17,40]. However, we introduce a novel method to compute the ratio of between-class distances to within-class distances. Finally, subjects control a designed telecar based on different paradigms using the optimal channels and features.

The structure of this paper is as follows: Section 2 describes two types of sEMG acquisition systems. Experimental methods about anticipants, experiment design, sensors placement are discussed in Section 3. Signal processing techniques including preprocessing, feature extraction, selection, and classification are explained in Section 4. Section 5 introduces four different control paradigms, followed by the results of offline analysis and real-time control in Section 6. Finally, Section 7 and Section 8 discuss and conclude with the strengths of the current work.

## 2. System Architecture

The entire circuit structure of two types of sEMG acquisition systems consists of four main parts: a power module, a signal-conditioning module, a signal-processing module, and a signal-transmission module. The power module provides required power and safety precaution for using in human recordings. The function of signal-conditioning module is to amplify and filter raw signals. Analog filtered sEMG signals are converted into digital signals in the signal-processing module. Finally, the signal-transmission module transmits these digital signals to PC. Our overall hardware design includes the requirements of low cost, low power, small size, human compatibility, and ease of programming and interfacing with standard computers. 

### 2.1. Offline sEMG Acquisition System

**Figure 1:**
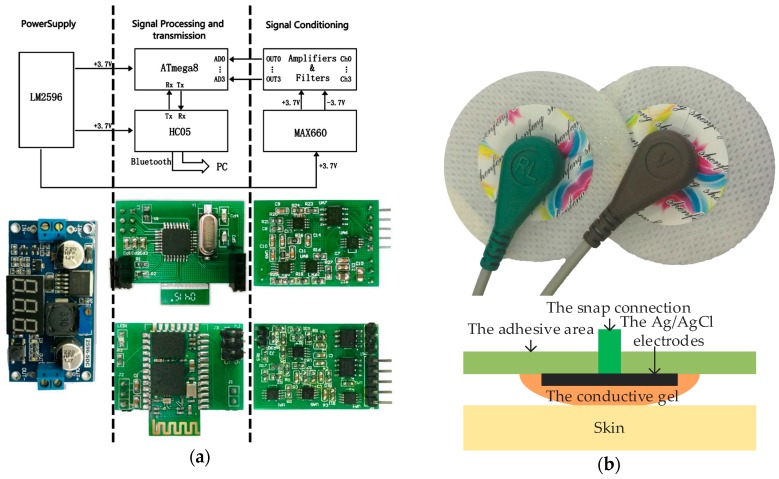
(**a**) The four-channel offline acquisition board; (**b**) The wet disc sensors and architecture.

We used disposable disc sensors in offline sEMG acquisition, as shown in Figure 1b. These sensors consist of Ag/AgCl electrodes, conductive gel, an adhesive area and a snap connection. Wet sensors with conductive gel ensure an easy conversion between ionic current and electron current, resulting in low electrode impedance up to few kilo-ohms [53]. Diameters of the Ag/AgCl electrodes, conductive gel, and disc sensors are 9, 15 and 34 mm, respectively. Two sensors, at a distance of roughly 30 mm, constitute a pair of bi-polar sEMG electrodes.

The sEMG signal has small amplitude and is severely distorted by electromagnetic interference. An approach to reduce the electrode-skin interference is to employ an amplifier with high input impedance. The acquisition system board presented in Figure 1a (32 × 22 mm^2^) includes a high-performance voltage follower (AD8626, Analog Devices, Norwood, MA, USA) as well as a differential amplifier (INA128, TI, Dallas, TX, USA) with very large input impedance. Together they largely reduce common mode interference (CMI) as well as improve the common mode rejection ratio (CMRR) and signal to noise ratio (SNR). We further reduce differential mode interference (DMI) with anti-aliasing and on chip digital filters. In both hardware and software solutions, the high-pass filter may have a 3 dB cutoff frequency of 10–20 Hz and the low-pass filter a 3 dB cutoff frequency of 400–450 Hz to avoid loss of information from the sEMG signals [54]. Therefore, a pass-band filter between 10 Hz to 450 Hz is designed using the AD8626. The sEMG signals are further passed through a notch filter at 50 Hz (UAF42, TI).

For the signal-processing module, we used an ATmega8 low-power 8-bit microcontroller (ATMEL, Microchip Technology, Chandler, AZ, USA), as the central processor and analog to digital converter. The supply voltage and reference voltage are both 3.7 V. The amplified and filtered signals (in the range of −1.8 V to 1.8 V) are then transformed to unipolar signals in a dynamic range of 3.7 V, sent to the analog-to-digital converter, and finally transmitted to a PC with Bluetooth UART module (HC05). We receive the signals at 1000 Hz sampling rate. They are further filtered (bandpass 10 Hz to 450 Hz as well as notch 50 Hz and its harmonics) and then stored via MATLAB.

The voltage convertor (LM2596, National Semiconductor, Santa Clara, CA, USA) including the thermal shutdown and current limit protection cells can provide the power of +3.7 V efficiently. The CMOS monolithic voltage converter chip (MAX660, Maxim, San Jose, CA, USA) produces a −3.7 V power to supply the negative voltage to dual-supply amplifiers.

### 2.2. Wearable sEMG Acquisition System

**Figure 2:**
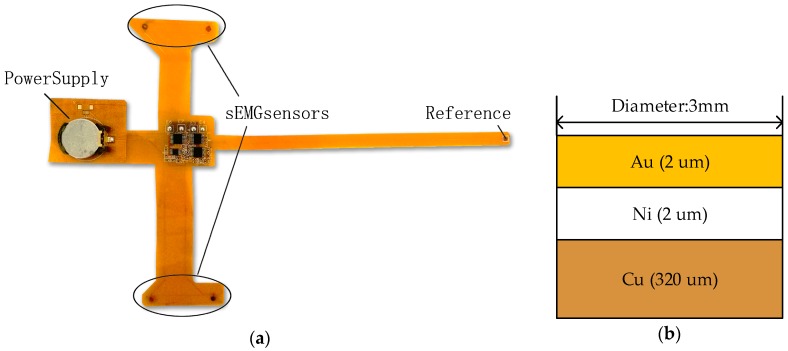
The two-channel real-time acquisition system. Subfigure (**a**) correspond to the acquisition board, and subfigure (**b**) correspond to the architecture of the metallic dry sensors.

The wearable sEMG acquisition system, shown in Figure 2a, is almost same as the offline system, with small differences. First, the FPC-based real-time system is more flexible, small-sized, lightweight, and low-cost compared to the PCB-based system. Therefore, this design minimizes noise pickup in sEMG recording stations, and allows for recording without additional pre-amplification steps. 

Second, in term of sensors materials, we designed two pairs of metallic dry sensors. This type of disc sensors is plated with copper on the top layer of FPC-based board. The next step is plating nickel and gold on the copper disc to stabilize contact impedance as shown in Figure 2b. Low electrode-skin impedance is critical for recording high-quality signals. The traditional solution is to gently exfoliate skin using abrasive gel or 75% alcohol. The diameter of each sensor is 3 mm and the fixed distance within a pair of sensors is 30 mm. The inter-electrode distance can be minimized further due to the smaller size of electrodes. Therefore, metallic dry sensors can fit an uneven skin for more precise applications.

Thirdly, the optimization of circuit structures is considered to satisfy demands of miniaturization and high-reliability for systems. The contact-impedance problem is a much more pressing problem for dry sensors compared with wet sensors [55]. Except for cleaning skins, another practice is to employ an amplifier with high input impedance. Therefore, we replaced the amplifier INA128 with a higher performance instrumentation amplifier INA2126 (TI). In order to minimize the system size, we replaced the AD8626 amplifier with a TLV4333 (TI). The TLV4333 contains a four-channel amplifier with an input filter to reduce both common mode interference (CMI) and differential mode interference (DMI). To combine the signal-processing and signal-transmission modules, the nRF51822 (Nordic, Oslo, Norway) is used as central processors because it integrates Cortex-M0 kernel, analog to digital converter (ADC), and Bluetooth 4.0 module. We used a 3 V button battery to power the real-time system.

Finally, wearable online systems are paired with a personal computer which performs more computationally intensive processing steps. These include independent component analysis (ICA), multiscale principle component analysis (MSPCA), specified features calculation, and prediction based on the trained model.

## 3. Methods

### 3.1. Subjects

Nigh able-bodied subjects (seven male and two female; mean age: 25.1; range: 21–31) participated in the data acquisition, all of whom had no prior experience with sEMG based HCIs and signed the consent form approved by the Academic Ethics Committee of Southeast University before experiments. All subjects are university-educated and non-smoking with no history of forearm muscle injuries and neurological disorders. Instructions for offline and online experiments were carefully explained and illustrated, and the first trial did not begin until subjects indicated full understanding of the required tasks. During all implementation processes, subjects sat motionless in a comfortable chair and rested their hands on a desktop.

### 3.2. Acquisition Setup

**Figure 3:**
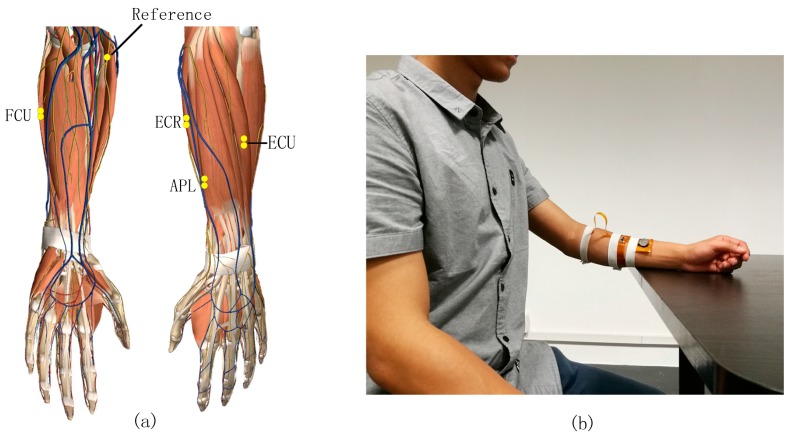
Sensor placement and implementation of the system. (**a**) Sensor placement in the offline analysis and the pairs of yellow points correspond to the bi-polar electrodes; (**b**) Real-time system setup for data acquisition.

In offline experiments, we selected four different muscle groups: flexor carpi ulnaris (FCU), extensor carpi radialis (ECR), extensor carpi ulnaris (ECU), and abductor pollicis longus (APL); as shown in Figure 3a. The activity of these muscles were measured via pairs of bipolar sEMG sensors during four wrist movements including wrist extension (WE), wrist flexion (WF), make a fist (MF), and rest (REST). The reference sensor was placed on the upper arm far from recording channels.

Figure 3b shows the real-time system setup. Four controlling states namely forward, backward, clockwise rotation and anti-clockwise rotation were mapped with four motions. Subjects can control the vehicle easily because the direction and rhythm of wrist movements correspond to commands of vehicle motions. We performed channel selection technique on data pooled from all subjects and found that the location with highest classification accuracy are ECU and ECR.

### 3.3. Experiments Protocol

Each subject was connected and sat for one complete recording period. The experimental period was divided into five offline sessions (1 h) and eight real-time sessions (0.5 h). In offline sessions, subjects maintained 1.5 s movements according to cues on the screen. Signals were recorded continuously and saved separately for each session. Within each session, subjects performed 40 individual motion split evenly and randomly ordered among these four movements. 

The same group of subjects attended real-time tests. We optimized channels, features, and classification parameters during offline sessions and utilized these optimal values for online processing. The telecar was controlled by four methods: the joystick paradigm, the fixed-moving sEMG paradigm, the channel-combination paradigm, and the state-machine paradigm. Each paradigm was repeated two times. The real-time analysis window was 125 ms with 20% overlap. The entire trajectory was a square. 

## 4. Signal Processing and Pattern Recognition

**Figure 4:**
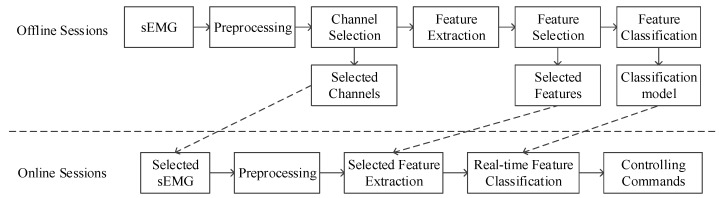
Flowchart of proposed processing framework.

All data processing was performed within MATLAB. The steps of signal analysis and the relationship between offline and online sessions are illustrated in the flowchart of Figure 4. In the offline phase, we used infinite impulse response (IIR) filters, ICA and MSPCA to de-noise sEMG signals. The feature extraction module includes time-domain, frequency-domain, and time-frequency-domain features computation. Feature selection refers to separability. The rank of single-channel accuracies selected optimal channels, and the EC and FD methods determined a uniform subset of features. Four machine learning algorithms (kNN, ANN, RF and SVM) were employed to classify features, and the best parameters and model were saved. In online sessions, same preprocessing approaches except a different segmentation method were adopted for signals from the selected channels. We then extracted the optimal feature subset according to offline sessions and utilized a classifier model to specify the control commands during online sessions.

### 4.1. Preprocessing

#### 4.1.1. Data Filtering and Segmentation

We employed an IIR Butterworth bandpass filter (10–450 Hz, with order 16) according to the previous works [54,56]. An elliptic notch filter and several bandstop filters were used to eliminate the power line interference at 50 Hz and its harmonics. There are two main sEMG segmentation methods: disjoint and overlapped methods [57]. In offline sessions, subjects maintained each motion in a 1.5-s task time, which had precise onset and offset boundaries. The task period was further divided into 3 periods—a transient period of onset (0–0.25 s); a one-second execution period (0.25–1.25 s); and another transient period. Feature values were extracted only from the one-second execution window segmented by the disjoint method with a predefined length. For example, four feature vectors could be extracted from the execution period with a 250 ms analysis window. Real-time analysis adopted the overlapped segmentation method. The size of sliding window was predefined with 20% overlap.

#### 4.1.2. Independent Component Analysis (ICA)

Subsequently, remaining artifact signals could be removed conveniently by applying ICA. For the general ICA model of sEMG, suppose we have *N* channels filtered sEMG signals *x_i_*, *i* = 1, …, *N*. Each channel has *N* independent source signals *s_i_*, *i* = 1, …, *N* and records different mixture of *s_i_*. Mathematically, the principle of mixing processes can be expressed as follows:

(1) x=A·s,

where *A* is the unknown mixing matrix, *x* and *s* represent the combination of *x_i_* and *s_i_* respectively. Then, the algorithm extracts a matrix with independent components (ICs) that recovers original sEMG signals when applied to the data set *x* according to:

(2) u=W·x,

where *W* called unmixing matrix equals *A*^−1^, and *u* denotes the sources (ICs). After performing an ICA, clean sEMG signals used in future processing are obtained by removing the ICs with artifacts and neglecting the corresponding column of *W*.

#### 4.1.3. Multiscale Principle Component Analysis (MSPCA)

The MSPCA algorithm was proposed by Bakshi [58] to merge the strengths of PCA with the benefits of the wavelet transform (WT). While PCA extracts the linear or nonlinear relationships among variables, WT extracts deterministic features and approximately removes the autocorrelation within measurements [59].

MSPCA has been applied for EMG [36] and ECG [60] signal modeling and de-noising. In terms of sEMG signals, the algorithm steps are: (1) The *j*-th column $x_{j}(t)$ of raw data are decomposed to its wavelet coefficients by WT. (2) The covariance matrix of each scale is computed along with the number of principle components separately from other scales. (3) The appropriate number of principle components is selected. (4) The combination of WT and PCA is used to reconstruct the de-noised signals.

### 4.2. Feature Extraction

Three main types of features—time, frequency and hybrid domains—have been used to classify sEMG signals for HCI [61]. These features are computed based on signals’ amplitudes (time domain), estimated power spectrum density (frequency domain) and time-frequency transformation (hybrid domain). The time domain features are most popular because of their computational simplicity. Time-domain features include mean absolute value, modified mean absolute value with the weighting window function (function (1)) and the improved weighting window function (function (2)), root mean square, variance, waveform length, Willison amplitudes, simple square integral, zero crossing, slope sign change, and histogram of sEMG. Frequency-domain features are mostly used to study muscle fatigue and to recognize movements. Widely used frequency-domain features include auto-regressive coefficients, median and mean frequencies. 

Time-frequency analysis, with its ability to represent time dependent frequency responses, has recently leveraged in the sEMG feature extraction. The most commonly used analysis method is discrete wavelet transform (DWT). We use the Daubechies 4 (DB4) wavelet because of higher classification accuracy and lower computation cost [62]. Average power of wavelet coefficients in each sub-band are extracted for evaluation of the frequency distribution. We also compute standard deviation of coefficients to evaluate changes in the distribution. Another time-frequency feature is power spectral density (PSD) of short time window Fourier transform. 

Table 1 lists features used in this study, their abbreviation and dimensions. In offline sessions, all 42 listed features were extracted for each channel. These features were then cascaded into a final vector with a dimension of 168 for four channels.

### 4.3. Feature Selection

The dimension of features extracted in the last section should be reduced before sending them to classifiers. Efficient feature and channel selection algorithms improve the prediction performance and provide less computational complexity. The common approach to evaluate selected features is estimating the rank of classification rate or the separability criteria [63]. 

Here, the optimal-feature selection combined the rank of separability values (SV) of each feature with classification rates by SVM. First, we obtained a feature subset with the highest accuracy for individual channel. The mean number of optimal feature subsets across all channels and subjects represented the size of features in real-time algorithms. Next step was to confirm the detailed common features. We calculated the summation of each feature’s SV via EC and FD methods. We then used the RFE method to compute and rank the frequency each feature was contained in optimal subsets. The selected features were determined by specific criteria according to these different methods. Ideally, different algorithms should obtain almost same optimal feature subsets.

#### 4.3.1. Entropy Criterion Based Feature Selection (EC)

A modified entropy based method is used to calculate separability values. The variance of features among different classes can provide classification information, and the entropy of features’ variance is a measurement of uncertainty. When the variance of different classes is close, it means that the specific class has little classification information and vice versa. Therefore, the entropy of variance measures separability of each feature. The definition is as follows:

(3) $$J_{i}=-\sum_{k=1}^{n}V_{k}^{i}\cdot \ln(V_{k}^{i}),$$

where *J_i_* denotes SV of the *i*-th feature, and $V_{k}^{i}$ denotes normalized variance of the *i*-th feature for the *k*-th class (totally *n* class). Within this method, smaller *J_i_* corresponds to the feature with larger variance entropy.

#### 4.3.2. Fisher Discrimination Based Feature Selection (FD)

The ratio of between-class and within-class distance could evaluate the extracted features’ separability numerically. The principle of this method is similar with the Fisher linear discriminant analysis (LDA) algorithm [64]. The SV is calculated by:

(4) $$J_{i}=D^{2}(a^{i},b^{i})/(D^{2}(a^{i},a^{i})+D^{2}(b^{i},b^{i})),$$

where *a^i^* and *b^i^* denote the *i*-th feature in class *a* and *b* respectively, and the function D^2^(*a*,*b*) is the mean Euclidean distance between all combination of different trials of two groups. The separability improves when the ratio increases. In multi-class (totally *n* class) separability analysis, we separate the problem into *n* two-class problems according to one-versus-all strategy [65]. The average ratio of these two-class problems is computed as the multi-class SV for each feature.

#### 4.3.3. Recursive Feature Elimination (RFE)

Compared with the mentioned methods offering the numerical evaluation of features, the RFE algorithm based on SVM outputs a list of ranked features. In detail, the RFE algorithm mainly contains following steps [66]. (1) Features and class labels are combined. (2) Training the model of SVM. (3) Computing the weight vector and rank criteria. (4) The feature with the smallest rank criteria is eliminated. (5) Steps (2)–(4) are repeated until only one feature is left. Finally, the algorithm outputs the feature rank list. The rank criteria is the squared coefficients *w*^2^ [67]. Importance of a feature is determined by the loss of the margin between classification boundaries when the feature is removed. The rank criteria is defined as:

(5) $$J_{i}=\underset{i}{\min}\left|w-w^{\left(-i\right)}\right|,$$

where *w* is the inverse of margins which means $w={\left\|w\right\|}_{2}$, and *w*^(−*i*)^ represents the *w* without the *i*-th feature at this SVM iteration. 

### 4.4. Classification

Although we quantify the separability of various sEMG features, it is still unclear whether they interact well with the classification process. Therefore, it is important to choose the best classifier for recognizing sEMG patterns. Here, four widely-used classifiers—kNN, ANN, RF and SVM—were Considered. We optimized classification parameters based on offline data (train) and then applied the optimal classifier to real-time data (test).

#### 4.4.1. k-Nearest Neighbor (kNN)

The kNN is one of the simplest learning methods that divides data into two or more classes. The kNN is frequently used for small training datasets, because it is easy to implement and has low computational cost. Inputs consist of the *k* closest training samples in the feature space. In the sEMG classification, the distance of *k* nearest neighbors from one another determines the label of test samples. Performance of the kNN depends on the selection of parameter *k*. Wan et al. tested the relationship between *k* and ten-fold CV accuracy [68]. When *k* is in the range of 3 to 10, the difference of accuracy is not huge. In this work, six nearest neighbors were selected to evaluate accuracies.

#### 4.4.2. Artificial Neural Network (ANN)

The ANN follows a learning method with self-learning capability [69]. However, because the network contains numerous parameters its training process is time-consuming. These parameters including thresholds of hidden layers and connection weights between layers. In the classification of four-motion sEMG signals, the ANN structure consisted of one input layer, one hidden layer and one output layer. The dimension of input feature vectors was *n*. The neurons of input, hidden and output layers were *n*, 2*n* and 4, respectively. The activation function was a sigmoid. We estimated parameters by the back-propagation algorithm to reduce the cost function and gradient [70]. Because of long training time, we used a five-fold CV to validate classification of sEMG data.

#### 4.4.3. Random Forest (RF)

The RF is a type of ensemble learning method. Although the design and computation are easy, it works better than other high-performance classifiers, such as SVM and ANN, in some applications [71]. In order to ensure performance of the RF, each base learner should have high precision. Simultaneously, to improve generalization ability of the RF, the diversity of base learners is guaranteed by two methods. The first method is to sample training data randomly as the input of each base learner. The second method is to choose the best decision feature from a subset of features (dimension: *s*) instead of from all features (dimension: *d*) for each node. The output is a final class voted by all base learners. In this study, the optimum number of base decision tree was 30 according to the research results of Gokgoz and Subasi [12]. The optimal feature subsets for nodes were determined as follows [72]:

(6) $$s=\log_{2}d.$$

#### 4.4.4. Support Vector Machine (SVM)

The SVM has high speed in calibration and classification of high dimensional sEMG features. The goal of this algorithm is to solve classification problem by finding maximal margin hyper-plane (***w***,b) to separate training data with a given set of labels. Briefly, a positive real constant α is computed by training data to determine parameters *w* and *b*. When using the test feature *f*, a label is assigned according to the decision boundary function, which is:

(7) $$g(f)=\mathrm{sign}(\langle w,f\rangle +b)=\mathrm{sign}(\sum_{j=1}^{m}\alpha_{j}y^{j}K(f,f^{j})+b),$$

where *f^j^* denotes the *j*-th trial (totally *m* trials) in training data with a corresponding label *y^j^*, and K is a kernel function including a high dimensional model. In this work, we chose the radial basis kernel function in LIBSVM [73]. Despite more than two movements present, the binary SVM was still used with one-versus-all technique. In offline sessions, ten-fold cross validation (CV) was used to assess classification accuracies and F-score.

## 5. Control Methods

**Figure 5:**
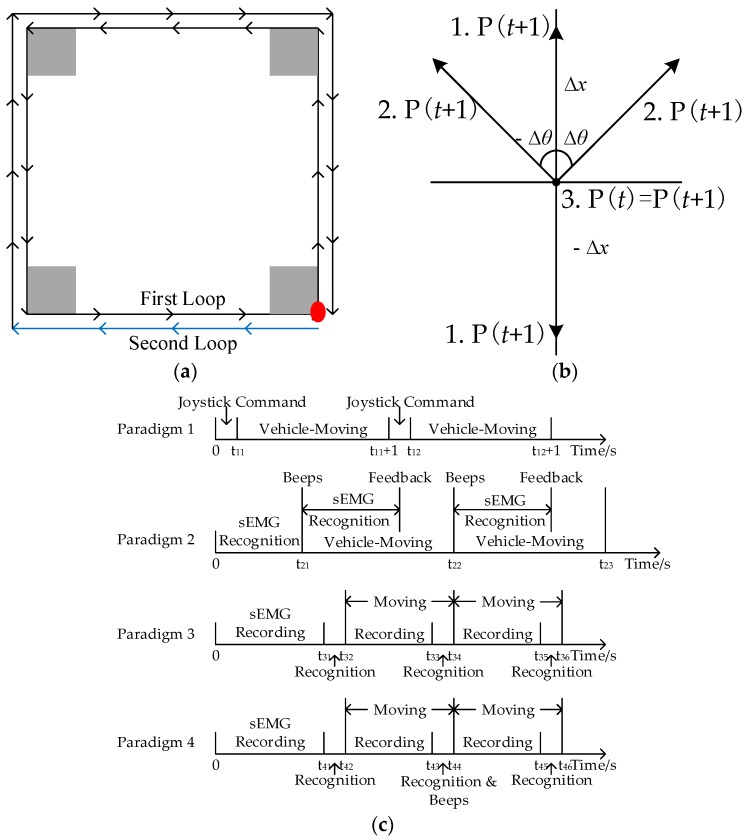
(**a**) Environment and obstacle maps. It contains the forward information (black vectors), backward information (blue vectors), starting point (red circle) and obstacle location (gray areas); (**b**) Three possibilities of the location update for Paradigm 1 and 2 (‘1’ denotes the straight-line movements, ‘2’ denotes the rotation movements, and ‘3’ is no movements); (**c**) The protocol for each paradigm. The onset of each real-time session is presented as the reference time point of 0 s.

Subjects can control a wheelchair—the final aim of sEMG-based HCIs—only when they achieve high performance in the telecar control with a pre-defined path. In real-time sessions, subjects controlled the designed toy vehicle with the wearable sEMG system to finish two loops in a square-loop environment with some simple obstacles. The length of each side was 40 cm. The vehicle was randomly positioned at any corner after the obstacle localization was completed. The moving and rotating speeds are set to a constant value of 12 cm/s and 0.25π rad/s, respectively. Figure 5a shows an ideal route to finish the loops and simple obstacle map. The differential distance of two loops in the figure is only for clear visualization. 

This section introduced four control methods. The response time of a real-time system should not introduce a delay that was perceivable by users, and the threshold was generally regarded to be roughly 300 ms [74]. Therefore, the real-time control in this work adopted a 125 ms window. There are two controlling rules: First, only one motion was classified at a time during the implementation. Second, in Paradigm 2, the classification result of newly acquired sEMG features combined with the last motion to confirm whether two consecutive windows were same. The protocol for each paradigm is shown in Figure 5c, and introduced as follows:

*Paradigm 1*: The vehicle moved to a direction according to the joystick command. The maneuverability is best for healthy subjects. 

*Paradigm 2*: Considering safety and a continuous control, the fixed-moving paradigm was introduced. In this paradigm, the next motion was determined during vehicle moving. Once the vehicle moved, subjects were prompted to start a motion with an auditory signal (beeps). After our system identified the motion, subjects received another auditory feedback and prepared for the next move. The epoch of sEMG data processing was 125 ms, and the same results of two consecutive epochs were considered as a valid control command. The HCI system would translate predicted classes into corresponding actions as described in Table 2. Suppose *P*(*t*) is the location at the time window *t*, and ∆*x* and ∆*θ* denote the position change in straight-line and veer directions. Then, the position at the next time window *t* + 1 could be updated as:

(8) P(t + 1)=P(t)±Δx, P(t + 1)=P(t)±Δθ, or P(t + 1)=P(t).

The equation reveals that the position at *t* + 1 has three possibilities as shown in Figure 5b: (1) Fixed ∆*x* determines the vehicle moves forward or backward by 12 cm. (2) Fixed ∆*θ* influences veer movements, which means the vehicle rotates 45 degrees clockwise or anti-clockwise. (3) If there is no movement commands, the vehicle waits and then stops. 

Because of the high separability of sEMG signals among different movements as well as the efficient auditory cues and feedbacks, this paradigm had a high degree of maneuverability in continuous HCIs.

*Paradigm 3*: Although the second paradigm has a good performance, some problems still exist. The main problem is about fixed moving periods, which leads to challenges for paths that frequently change directions. Another problem is that when no continuous same results come, the delay may still exists. 

To overcome these problems, we proposed the channel-combination paradigm with 125 ms window. The same results from both selected channels determined moving directions during recognition periods (i.e., t_31_–t_32_, t_33_–t_34_ and t_35_–t_36_ in the figure). Table 3 shows control methods in this paradigm. The sEMG recording for the next process was synchronous with the vehicle moved. For example, the recording time was from t_32_ to t_33_ and the vehicle-moving period was from t_32_ to t_34_. The respond time is short in this paradigm, but subjects could not stop by their autonomous motions in the four-state control, which is a hidden danger for patients.

*Paradigm 4*: The state-machine-based control paradigm could increase the functionality [48]. Five-dimension control could be achieved by four motions in this paradigm as shown in Table 4. The REST state was a switching of straight-line and rotational movements. The protocol was similar with Paradigm 3 in Figure 5c. The initial state was straight-line movements, and the detailed control method was as following. Recognition results from t_41_–t_42_ determined moving states in t_42_–t_44_. Upon the REST state appeared (e.g., the period of t_43_–t_44_), an auditory beeps was offered and the mode was switched to rotational movements. Consequently, another REST state set back the mode to straight-line state. A critical control rule was the mode could not be changed unless at least one motion was implemented. Subjects can stop control by keeping fisting.

## 6. Results

Our objective is to choose and use effective features for the sEMG-controlled vehicle with wearable HCI designed by our group. We present four steps: first, we validate the feasibility and performance of our proposed hardware and filters. Second, the rank of classification accuracy picks two channels. Thirdly, optimal feature subsets and multi-class recognition rates are computed by the proposed feature selection algorithms, and compared with the RFE method. Finally, we generalize findings through comparing different control paradigms, and investigate whether the selected common channels and features are applicable to online sessions.

### 6.1. Acquisition System Testing 

Tests of this part are to verify the feasibility of acquisition systems and preprocessing methods. The results show that real-time high-quality signals can be transmitted to computers and saved within the Bluetooth communication distance. 

#### 6.1.1. Hardware Evaluation

**Figure 6:**
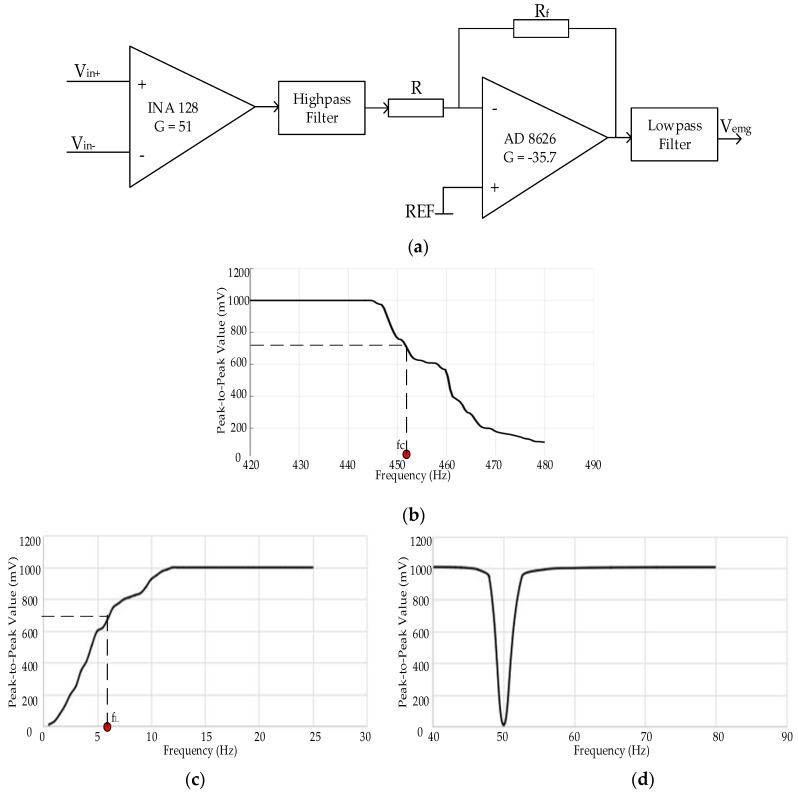
(**a**) is the system electrical schema of amplifiers and filters, (**b**–**d**) are the amplitude-versus-frequency curves of low-pass filter with the cut-off frequency fc, high-pass filter with the cut-off frequency f_L_, and notch filter of 50 Hz, respectively.

Signal amplifiers and filters are the main components in acquisition systems. Two stages of amplifiers were used to avoid effects on the signals’ bandwidth when the gain of one stage amplifier was too large. The first stage amplifier has a large input impedance, and the gain is 51. The second stage is an inverting amplifier with high gain (−35.7), gain bandwidth product (GBP) and CMRR. Therefore, the total gain for amplification of raw sEMG signals is −1821 as shown in Figure 6a.

The acquisition systems contain a low-pass filter of 450 Hz, a high-pass filter of 10 Hz and a notch filter of 50 Hz. We validated designs of filters and circuit components in the FilterPro (TI) instead of via manual derivation process. The low-pass and high-pass filters are four-order and two-order Butterworth structure with the Sallen-Key topology, respectively. In addition, a 50 Hz notch filter is integrated in the UAF42.

Frequency responses of filters were measured by applying a 1 Vpp sinusoidal signal logarithmically generated by a function generator. Figure 6 depicts the amplitude-versus-frequency curves. The frequency range of testing signals is from 1 Hz to 480 Hz. From Figure 6b,c, acquisition systems show a flat operation on the edge of frequencies of interest (10–450 Hz). The selection and parameter errors of resistors and capacitors resulted in the real cut-off frequency range of on-chip filters is from 6 Hz (f_L_) to 451 Hz (fc). In Figure 6d, we show effects of the notch filer, the interference of 50 Hz has been reduced.

In order to evaluate performance of the recording hardware, we computed the SNR as follows:

(9) $$SNR=10\log_{10}\left(\frac{A_{m}^{2}}{A_{r}^{2}}\right),$$

where *A*_m_ is the maximum RMS amplitude of continuous strained muscles signals and *A*_r_ is the maximum RMS value of noise when a muscle is not activated. SNR of the offline and online acquisition systems are 47.42 dB and 54.09 dB, respectively. The slightly higher SNR in the wearable system can be attributed to the optimized circuit structure and selection of high-performance chips.

#### 6.1.2. Test on the Digital Signal Preprocessing

To improve SNR of systems and quality of sEMG signals, the preprocessing module contains several digital filters, including band-pass filter in the frequency range of interest (10–450 Hz). When adopting dry sensors, significant 50 Hz noise pickup interfered with signals. In order to eliminate this power line noise, we used a combination of analog and digital filters including the elliptic notch and band-stop filters. 

**Figure 7:**
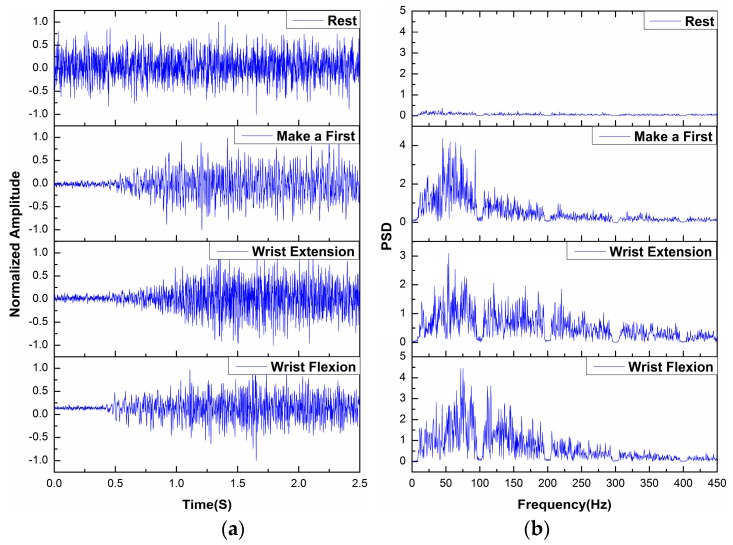
Time-frequency verification of different movements: (**a**) Normalized amplitudes in time domain; (**b**) PSD in frequency domain. (The names of four movements were represented to their abbreviations in the following analysis)

The features in time and frequency domains were extracted. Therefore, this section presents the time-domain and frequency-domain verification. Figure 7a shows time-domain signals at the APL after preprocessing and normalization. An increased amplitude appears after zero to one second in the last three movements, because subjects keep rest in this period. Because of the normalization of each state, amplitudes of signals within 0–1 s in the last three plots are not similar to amplitudes of the REST state. The differences among different movements are clear. The REST and WE states have respectively the smallest and largest amplitudes. The time-series for the WF and MF tasks closely resemble each other. In detail, the MF state has slightly higher amplitudes than the WF state.

The power spectral density (PSD) was estimated for each trial. Averaged PSD zoomed in the range of 0–5 is depicted in Figure 7b for the time span from 1 to 2.5 s. The MF state has the highest mean PSD, followed by the WF, WE and REST states. In detail, the MF and the WF states have the highest PSD in sub-bands of 10–105 Hz and 105–195 Hz, respectively. Relevant frequencies of all movements are between approximately 10 Hz and 450 Hz. Neural information plateaus around 40–95 Hz, and the range of interest extends to 40–195 Hz for the WF state. Then, the PSD diminishes slowly as the frequency increased to 450 Hz. Mentioned notch and bandstop filters can eliminate the 50 Hz and its harmonic. SNR is improved to 61.47 dB and 68.91 dB for these two acquisition systems, respectively. 

### 6.2. Channel and Feature Selection

According to previous studies, longer time windows would not have significantly improved prediction accuracy [43,74]. All sEMG signals during training were analyzed in non-overlapping windows as mentioned in Section 4.1.1. Each motion modality could extract 160 feature vectors with 250 ms window. If the window length decreased to 125 ms, training sessions contained 1280 feature vectors. To ensure classification performance and reduce training complexity, the window length was 250 ms in the channel and feature selection. 

#### 6.2.1. Channel Selection

Although we use only four pairs of sEMG channels, it is still necessary to minimize the number of channels to make systems more mobile and easier to maintain. We rank single-channel classification accuracies using all features. Table 5 shows accuracies of each channel across all nine subjects. 

We then select channels located on the ECU and ECR according to Table 5. Features from the channel ECU achieve the best classification accuracy equal to 97.45%, followed by the channel ECR and FCU reaching 96.55% and 95.00%, respectively. The channel FCU is best for recognizing between the REST and motions states (i.e., the first three pairs in Table 5). The channel ECU provides the best accuracies compared among different motion states (i.e., the last three pairs in Table 5). When comparing all motion pairs, first three pairs have higher accuracies than last three pairs. The REST and MF pair obtains the best performance for all channels. Furthermore, the MF and WE pair has the highest distinction among last three pairs. 

**Figure 8:**
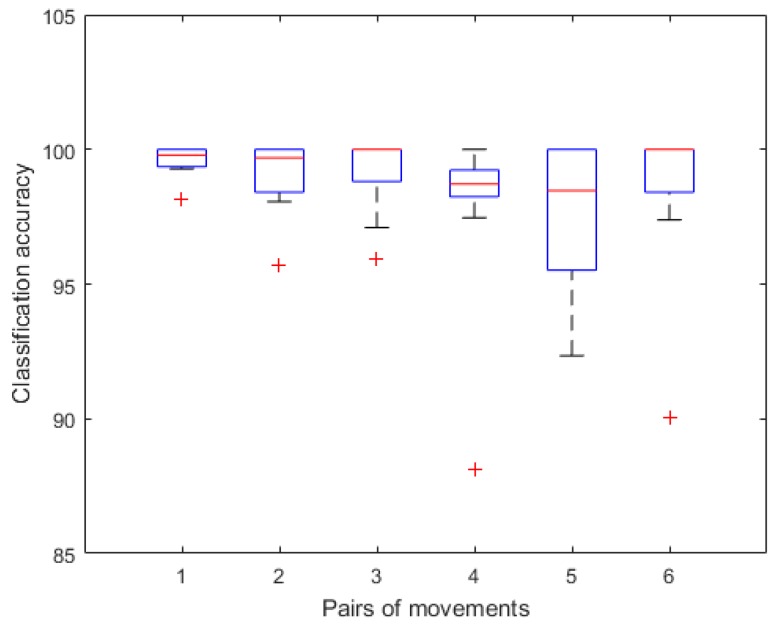
Boxplot for EMG tasks classification accuracy with ECU and ECR. The horizontal axis represents the different pairs of movements (1: REST vs. MF, 2: REST vs. WE; 3: REST vs. WF, 4: MF vs. WE, 5: MF vs. WF, and 6: WE vs. WF).

For each subject, we divided sEMG features from these two channels into training and testing sets by ten-fold CV to estimate mean classification accuracies of different pairs of movements as shown in Figure 8. Accuracies of one subject are lower, which are marked as outliers in the boxplot. Mean accuracies of the first three pairs are 99.56%, 98.99% and 99.12%, respectively. Classification results of the last three pairs are more than 97%. Especially for the third and sixth pairs, median accuracies reach 100%. Above all, compared with single-channel analysis, the selected-channel performance is not significantly improved in recognizing the rest state with other movements. However, channel selection improves accuracies of the last three pairs by 3.75%, 6.33% and 2.80%, respectively.

#### 6.2.2. Classification Performance between Each Two Motions

This part tests the single-channel separabilty between each two movements, and investigates the feasibility of feature selection methods. Proposed EC and FD methods ranked features between two motions. Classification rates were performed as features increased from 1 to 42 to determine the best combination of feature space. Mean classification accuracies (MCA) with optimal feature numbers (OFN) across all subjects were evaluated by LIBSVM in the proposed two situations, and compared with the RFE method. 

Table 6 lists MCA with OFN at the channel ECU. Classification accuracies are above 97% for all pairs except for the MF and WE pair. On average, accuracies are slightly higher with lower numbers of features opting for the FD and RFE based feature subsets. When comparing classification performance among different pairs, first three pairs obtain larger MCA with less OFN. Almost same accuracies between the REST and motions states reach about 98% by three feature selection methods. For last three pairs, average accuracies in the range of 94.72–98.16% and 94.68–98.15% are achieved by the FD and RFE methods compared with the range of 94.52–98.06% by the EC method. 

One-way analyses of variance (ANOVA) are used for statistical analysis. The factors for analysis are six pairs of motions and three feature selection algorithms. (1) According to Table 6, the OFN is influenced by different pairs (*F*(5,156) = 14.416, *p* < 0.001) as well as three algorithms (*F*(2,159) = 7.641, *p* = 0.001). Post hoc tests of the influence of pairs show that first three pairs use significantly small feature subsets compared to last three pairs, but no differences are found within these two groups. Post hoc tests also show that the FD and RFE methods differ significantly from the EC method (*p* = 0.002 and *p* = 0.003, respectively), indicating the EC method uses more features to reach the optimal accuracy. There are no differences between the FD and RFE methods (*p* = 0.991), because these two algorithms are both based on classifiers learning. (2) The MCA is also affected by pairs (*F*(5,156) = 8.716, *p* < 0.001), but do not show reliable relationship with algorithms (*F*(2,159) = 0.006, *p* = 0.994). Post hoc comparisons reveal that the MF and WE pair has significantly lower accuracy than other pairs. All other pairs have no differences within each other except for comparing the first and fifth pairs (*p* = 0.039).

**Figure 9:**
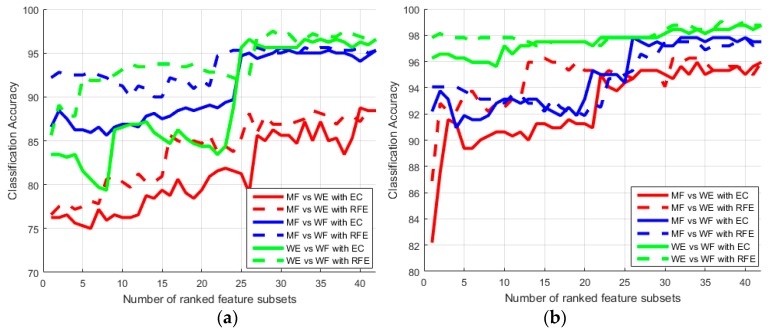
Comparisons of the RFE and EC methods. Evolution of classification accuracies was computed with feature subsets increasing according to different separability ranks: (**a**) classification accuracies of three pairs of motions for subject S6; (**b**) Same results for subject S7.

Furthermore, Figure 9 shows classification accuracies of three pairs of motions (i.e., the last three pairs in Table 6) as features increases by the EC and RFE ranks. The classification accuracy increases as the feature space increases. We assert that this is due to insufficient information provided with small feature subsets. However, when the feature size exceeds OFN, the accuracy remains high and then begins to decrease due to over-fitting. It illustrates one reason why feature selection is necessary. When number of selected features is less than 25, the RFE method performs better than the EC method. Then, performance of these two method reaches the same level. Figure 9a plots the best recognition rates of subject S6 could be improved to 89.38%, 95.63% and 97.5% with 36, 23 and 31 features picked by the RFE method for mentioned three pairs, respectively. As shown in Figure 9b, subject S7 uses the EC method to select 33 features to yield 96.25% accuracy comparing the MF and WE states, to select 37 features to yield 98.13% accuracy comparing the MF and WE states, and to select 33 features to yield 98.44% accuracy comparing the WE and WF states. 

#### 6.2.3. Feature Selection

The feature selection analysis is as follows. First, the dimension of optimal features is determined by single-channel analysis of ECU and ECR. This step combines feature selection and classification processes. Mean optimal feature numbers across all channels and subjects are 31, 23.3, 32.8 for the EC, FD and RFE methods, respectively. Then, the focus in this section is to identify common features from these subjects for future application. The advantage is that under repeated use, limitation to specified features reduces training and processing times. Two weighting methods were used to select specified features derived from the SV rank of different feature selection algorithms. 

The weighting methods based on proposed EC and FD methods belong to quantitative weighting methods because they have detailed numerical evaluation. The single-channel SV of each feature is normalized to the range of 0 to 1, and averaged across all subjects. Then the separability criteria is the summation of all single-channel SV. Detailed features are determined by considering the 1st–31st and 1st–23rd features of EC and FD methods respectively according to their SV ranks. Table 7 shows total selected features from mentioned two feature selection algorithms, as well as the separability value of each feature. For the FD method, improper amplitude thresholds lead to the exclusion of WAMP1 and entire HEMG features except for HEMG1. All frequency features and the STFT are also neglected. Removal of APWC_D4 to APWC_A6 and SDWC_D5 to SDWC_A6 indicates a low effect of low-frequency components. For the EC method, features in the time domain including ZC1, WAMP4, WAMP5, HEMG1 and HEMG2, as well as all frequency features are eliminated.

The RFE method can indicate whether an individual feature is within the optimal subset with 1st–33rd features for a particular subject. The frequency with which each feature occurred among the top 33 features, across all subjects and channels, is considered. These frequencies are sorted in a descending order. Table 7 presents the top 33 features and their frequencies marked as T_32_. Because we select two channels for nine subjects, the highest time should be 18. The results show that seven features form the best feature combination for this method. These features include variance, the first and third thresholds for Willison amplitude, and average power of the wavelet coefficients in the 1st–4th sub-bands. In contrast, two dimensions of the AR coefficients never enter top 33.

From the table, the optimal feature subsets with qualitative and quantitative weighting analysis indicate that time-domain and time-frequency-domain features lead to a better separability performance than frequency-domain features. 

### 6.3. Classification Performance

Four amplifiers (kNN, ANN, RF and SVM) and three analysis windows (125, 250 and 500 ms) were compared in this section.

#### 6.3.1. Comparisons of Feature Subsets and Classifiers

Table 8 summarizes classification results for different feature combinations. In this study, seven different feature subsets are classified by four different algorithms such as kNN, ANN, RF and SVM. Each classifier is trained and tested with data from the same subject. Bold numbers in Table 8 indicate the best classifier for each feature subset. RF and ANN classifiers perform better for all subsets. RF with FD-based features ranks first at 96.77%, followed by ANN at 96.67%, SVM at 95.40% and kNN at 94.41%. In classification of EC-based features, ANN provides the superior accuracy with 96.74%, and RF ranks second with 96.66%. SVM gives 95.37% and kNN is with 94.73% ACC. All classifiers deliver above 94% accuracies after feature selection. 

As shown in Table 8, classification performance of EC-based and FD-based features almost have no differences. Both of them are slightly better than RFE-based features and single-type features. Compared among single-type features (RMS, MAV, APWC and SDWC), the wavelet coefficients have better classification accuracies than RMS and MAV. The reason is that features of sEMG signals after time-frequency preprocessing offer a better classification precession [75]. Above all, smart combinations by feature selection methods provide more accurate features. 

F-Score is another index to evaluate classification performance calculated by the formula:

(10) $$F\text{-}\mathrm{Score}\;=\;\frac{2\;\times \;\mathrm{TP}}{2\;\times \;\mathrm{TP}\;+\;\mathrm{FP}\;+\;\mathrm{FN}},$$

where TP, FP and FN are the numbers of true positives, false positives and false negatives in the confusion matrix, respectively. ACC and F-Score are close to each other, which indicates all classifiers achieve reliable performance on these feature subsets. With EC-based features, RF obtains 96.66% ACC and 0.9656 F-Score. The F-Score of FD-based features classified by RF is 0.9669, which is coincident with 96.77% ACC. It is also the case for other classifiers.

For statistical analysis of classification accuracy, different feature subsets and classifiers are the factors. The ANOVA reveals significant effect of feature subsets (F(6,245) = 14.323, *p* < 0.001) and classifiers (F(3,248) = 2.990, *p* = 0.032). However, the two factors interacted missed the 5% criteria (*p* = 0.677). (1) From post hoc analysis, the feature subsets could be divided into two groups. The first group contains feature space selected by algorithms. The second group is all single-type features. The two groups differ significantly (*p* < 0.001), but no differences appear within each group (*p* > 0.5). The results demonstrate that performance of feature selection algorithms is significantly better than single-type features. (2) In view of different classifiers, RF is significantly better than kNN (*p* = 0.036) and marginally better than SVM (*p* = 0.109). Furthermore, ANN has almost similar performance with RF (*p* = 0.796).

#### 6.3.2. Comparisons of Analysis Window

**Figure 10:**
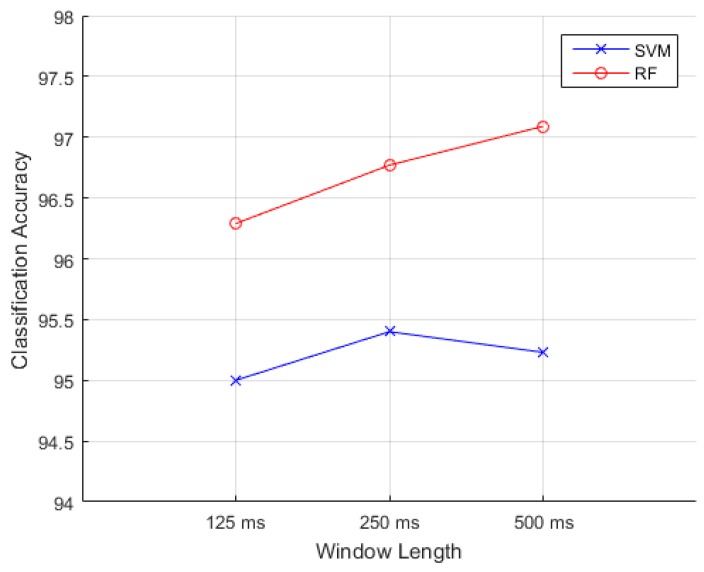
Effect of window length on the classification accuracy. The classification accuracies are averaged over all nine subjects.

Figure 10 shows the effects of analysis window length and accuracies. The mean accuracies are calculated by RF and SVM classifiers with the FD-based feature subset. RF performs better with these three epochs. Mean classification accuracies are 96.29%, 96.77% and 97.09% for the 125, 250, and 500 ms windows, respectively. The difference is non-significant (*p* = 0.744). Statistical analysis implicates that when shortening window length to 125 ms, the accuracy is not deteriorated. The advantages of adopting shorter windows are low computational cost and little storage space. Moreover, it is important with regard to the real-time classifier. 

#### 6.3.3. Comparisons of Confusion Matrices

**Figure 11:**
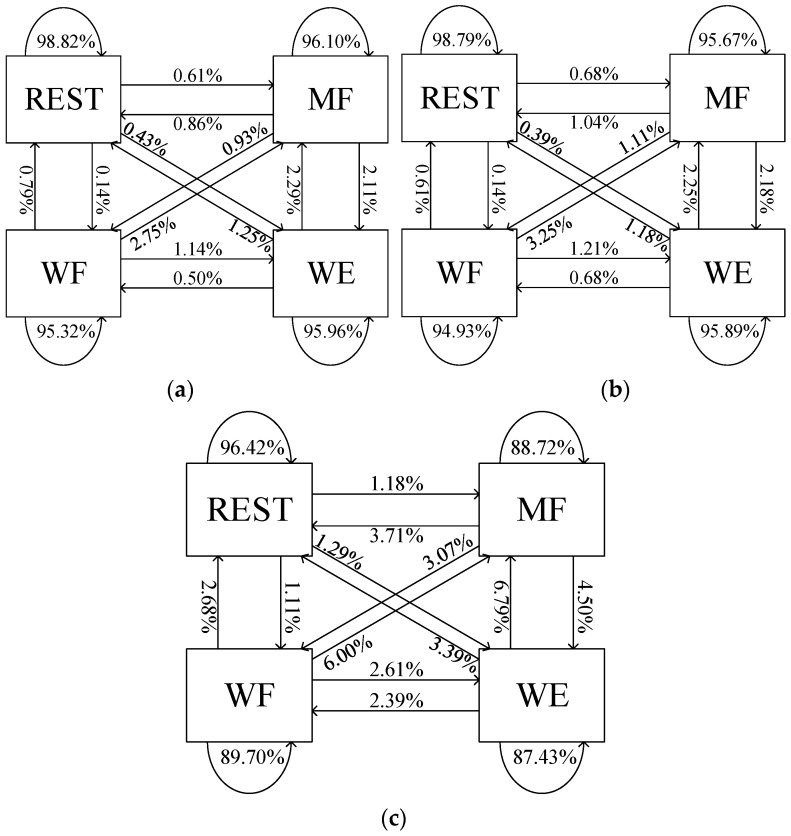
Recognition rate of the RF and confusion graph of different feature subsets: (**a**) the FD-based features; (**b**) the RFE-based features; (**c**) the APWC features. The numbers denote the percentage of samples in the class (arrow tail) classified as the class (arrowhead).

Each motion indicates a detailed command in the online system. Therefore, we structure the confusion matrix of each modality to investigate results of parameters and model selection. Figure 11 shows the recognition performance of FD-based, RFE-based and APWC feature subsets, respectively. 

These features are extracted from 125 ms windows, and classified by RF. The FD method (REST: 98.82%, MF: 96.10%, WE: 95.96% and WF: 95.32%) is slightly better than the RFE method (REST: 98.79%, MF: 95.67%, WE: 95.89% and WF: 94.93%). The feature selection process is helpful to classify all states compared to single-type APWC features (REST: 96.42%, MF: 88.72%, WE: 87.43% and WF: 89.70%). The REST modality achieves the best recognition performance as sEMG amplitudes of keeping rest and moving have large differences shown in Figure 7. Whereas, the MF, WE and WF modalities are misclassified to others, especially for the MF and WE modalities. These observations are in line with the results in previous sections. They indicate that assessment of multi-class recognition is feasible with proposed feature selection methods. Our proposed FD method can improve the prediction performance and reduce the feature numbers compared with the conventional RFE method and single-type features.

### 6.4. Online Evaluation by Wearable EMG-based HCI

Each subject performs eight online sessions according to four separate paradigms. The selected features extracted from optimal channels and the trained RF model are opted for online sessions. Averaged recognition rates (RR) and travel time (TT) of each paradigm for all subjects are recorded and referred to Table 9. In Paradigm 2, moving directions offer labels for classification. Therefore, the RR equals to classification accuracies. However, in Paradigm 3 and 4, subjects control the vehicle according to their thoughts. Here, the RR in Paradigm 3 denotes success rates of recognizing classification results of both channels are same. Since it is hard to define which motion is right in Paradigm 4, the RR is not presented in Table 9 as a criterion. 

The results illustrate all subjects are able to complete these online paradigms with acceptable accuracies and travel time. The TT in last three paradigms is close to the joystick paradigm (Paradigm 1). Averaged time of Paradigm 1 to finish the loops is 45.18 s. In the fixed-moving paradigm (Paradigm 2), subjects can accomplish two sessions within the mean time of 46.02 s with 95.01% RR. Paradigm 3 and 4 increase the control time to 49.06 s and 48.08 s, respectively. The increment is achieved that when two channels have different classification results in Paradigm 3, the vehicle stops and waits. Transience pauses also happen in mode switches during Paradigm 4. Although Paradigm 3 and 4 are more sophisticated, these paradigms are closer to daily life. In Paradigm 2, only one command could make the vehicle move a fixed distance. However, in Paradigm 3 and 4, subjects decide each minor motion by their own ideas. Compared these two complex paradigms, subjects use less travel time in Paradigm 4. The reason is that success rates are lower to ensure both channels have the same class for Paradigm 3. S3 completes all sEMG-based sessions using the shortest time with the highest RR. On the contrary, S8 performs the worst.

The rough relationship between the TT and RR in Paradigm 2 and 3 is that more TT uses, less RR obtains. However, there are some special situations in detail. For example, the TT of S9 is similar with S5 (46.59 s vs. 46.60 s in Paradigm 2), but S5 has higher RR (96.00% vs. 91.77% in Paradigm 2). S9 can send out control commands before the end of vehicle-moving periods, although he misclassifies some motions. The performance decreases sharply controlling Paradigm 3 for a small number of subjects. The RR reduces 9.37% and 7.74% for S2 and S6, respectively. According to the offline and online analysis, performances of two selected-channels have some differences. For S2, accuracies of ECR and ECU are 90.76% and 96.80% in the offline analysis. The problem is solved in Paradigm 4 to a certain extent because of using combined features from both channels. The real-time RR obtained in Paradigm 2 is slightly lower than offline sessions. A main reason is the states with high accuracies are less than offline experiments. For example, Paradigm 2 needs only four backward commands controlled by the REST state which has 98.82% offline RR.

The results of statistical analysis illustrate that the travel time shows a significant effect of paradigms (*F*(3,68) = 14.149, *p* < 0.001). Post hoc tests reveal the TT of Paradigm 1 is significantly shorter than Paradigm 3 and 4 (*p* < 0.001), but it has no difference with Paradigm 2 (*p* = 0.607). Subjects use slightly less time in Paradigm 4 compared with Paradigm 3 (*p* = 0.548).

**Figure 12:**
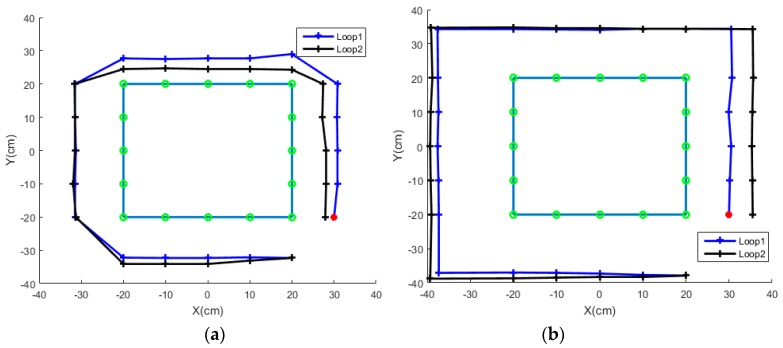
Rectangular route results for sEMG based control by different subjects: (**a**) path traced by S3; (**b**) path traced by S8.

The route tracking performance of two subjects for the rectangular route is provided in Figure 12. Position measurements are taken when the vehicle reaches a certain position as green circles in the figure. During the online implementation, rotational movements are more difficult than straight-line movements for most subjects. The difference between S3 and S8 is related to two control methods facing a turn. A group of subjects turns a degree, moves forward and adjusts the direction for the next straight-line motion, as shown in Figure 12a. The other group of subjects moves forward for an enough distance and makes an approximate 90 degree turn at the corner, as plotted in Figure 12b. The first group needs short path length, but also needs to change modes three times. After turning, the vehicle could move in a straight line without too many fluctuations.

## 7. Discussion

The purpose of this work was to design and demonstrate a type of sEMG-based HCI. The optimal combination of sEMG feature selection and classification methods is found and applied for online telecar control with the wearable acquisition system. The results demonstrate that the system with selected channels and features could achieve the classification accuracy and F-score above 90% in both offline and online experiments. This study provides potentials that patients with little motor ability could control the actual wheelchair with our system and algorithms.

### 7.1. Wearable EMG-Based HCI System Design

sEMG monitoring systems are suitable for wearable wireless applications that require small size, excellent mobility, low power consumption, and high transmission rates [76]. The most common systems were based on rigid PCBs [44,50,77]. The work studied by Kundu [50] proposed an EMG acquisition system equipped with a 7.4 V Li-ion battery, and then data were transmitted to computers via USB. Youn et al. [77] proposed a wireless sEMG system, whose size was 37 × 17 mm^2^, with a Bluetooth transmission module. In the design of another system, a 3.7 V Li-ion battery provided power to the system with a size of 34 × 25 mm^2^ [44]. Data were sent to a PC through a wireless module pair. Our proposed PCB-based system had a slightly smaller size (32 × 22 mm^2^) and the power was ±3.7 V. The main problem of these systems is mobility. Because the PCB-based systems still have connection wires between systems and sensors, they require complex fixation. Another problem is the sensor material. Although disposable sensors are convenient, they did not provide good performance in the accurate control on uneven skins due to the large distance between electrode pairs. 

To overcome these problems, we implemented all systems on the FPC with embedded metallic sensors. Flexible dry sensors based on the FPC substrate achieved comparative performance with standard wet Ag/AgCl sensors [78], and were approved for clinical applications. FPC lines connected the signal-conditioning and signal-processing modules designed on the PCB to transmit signals and power [79]. Here, dry gold-plated copper sensors were used and the inter-pair sensor spacing was set at 12 mm. The fixation distance between sensors pairs was 30 mm and could be adjusted as needed.

SNR of a system could influence the signal quality. Phinyomark et al. [80] demonstrated the relationship between classification accuracies and SNR. Different white Gaussian noises were added to make the SNR varied from 20 to 0 dB. When the level of SNR noise reached 20 dB, accuracies were close to clean signals. SNR of standard-wet and FPC-based sensors were 18.1 dB and 20.2 dB [78]. SNR of the system designed in Youn’s work was 59.06 dB [77]. SNR of our proposed PCB-based and FPC-based systems were 61.47 dB and 68.91 dB, respectively. 

### 7.2. Feature Selection and Classification

Other studies have recognized several sEMG patterns to different applications such as motions/hand gesture recognition, prosthesis control and diagnostic decision. To allow comparison of our findings with these literatures, we list methods, classification results and applications in Table 10. The averaged ACC of our paper is best. 

Efficient features selection algorithms could exclude many irrelevant and redundant features to provide higher performance. Nevertheless, the methods were not mentioned in some studies in Table 10. Fang et al. [32] just mentioned RMS was one of the most important sEMG features because of lower computational cost and decent performance. Another study used MAV for the same reason [44]. The optimal features should be extracted by some criteria. Tosin et al. [42] demonstrated that RFE was a powerful feature selection algorithm. However, the output was a list of ranks in separability without detailed values. Then, quantitative feature selection methods including the Davies-Bouldin index [82], RES [81] and Fisher Criterion [40] were introduced. There are two problems in these methods. First, success rates of these methods are not high enough (Table 10). The second problem involves the optimal number of features. Huang et al. [82] used a feature subset selected by the Davies-Bouldin index to obtain 85% and 71% classification rates in offline and online tests, respectively. In Lee’s work, the authors tested classification performance fixing the numbers of feature subsets to 100 and 150 [40]. In others, the feature selection process including CV of classifiers was complicated [81]. In our paper, the modified EC method was proposed because of low computational cost. We also combined Euclidian distances with the Fisher’s discriminant to obtain the modified FD method. The accuracies of these two methods were 96.66% and 96.77%, which were slightly better than the conventional RFE method and other feature selection methods in the table. The average number of features to receive the best accuracy for each selected channel across all subjects was defined as the optimal feature number. This method was more reasonable than Lee’s work [40], and easier than Srisuwan’s work [81].

### 7.3. The Online Performance

Since the final target of our systems is for a wheelchair control, the performance of smart wheelchairs is compared and discussed in this section. Delicate motions of the upper limb controlled joysticks-based smart wheelchairs [83], but they are not capable for patients with complete or partial loss of muscle activities. The EEG-based [83] and EOG-based [7] wheelchairs with automated navigation systems were proposed. In Huang’s work [7], subjects could control the wheelchair to finish all tasks within 227 s and 277 s by joysticks and EOG signals, respectively. The recognition rate for healthy subjects was 91.7%. The main challenge was to decrease misclassification rates of unwanted blinks or rotational motions of eyes. In Zhang’s work [84], the destination selection was fast, but the critical problem was subjects needed 4.5 s to stop control. 

The sEMG-based control method was considered in this work, because sEMG signals could achieve higher accuracies and use in long-term applications. The fixed-moving paradigm could improve safety. The average time was 46.02 s, which means each vehicle motion including the sEMG-recognition and vehicle-moving periods cost 1.05 s. The waiting time was much shorter than the same method in an EEG-based wheelchair [85]. 

According to Kucukyildiz’s work [86], the fixed-moving control paradigm had challenges for paths with frequently directional changes. Their work used very short analysis window (50 ms) for the sEMG control. Englehart et al. analyzed the effects of analysis window length upon classification accuracy [74]. The results showed that the best performance is with 32 ms analysis window with a majority vote decision. There was no differences when the window length ranged from 32 ms to 256 ms. However, in single-window analysis, the accuracy degraded rapidly with decreasing analysis window length. According to this work, the real-time processing window is 125 ms in our work.

To improve control’s continuity, the channel-combination and state-machine paradigms were introduced. The travel time of controlling by joysticks was 45.18 s. Subjects used 49.06 s and 48.08 s by these two continuous sEMG-based paradigms. The accuracy of Paradigm 2 was 94.79%. The recognition rate of motions was 91.77% in Paradigm 3. The same comparisons were shown in Kundu’s work [50]. The travel time of a designed wheelchair was 67.18 s and 72.88 s for joysticks and sEMG signals. The real time recognition accuracy was 90.58%. Despite the moving speed was lower and the path length was shorter in our work, the real-time results were acceptable.

The trend of sEMG-based HCIs is to increase the degree-of-freedoms. Maeda et al. designed an omnidirectional wheelchair with four-channel sEMG signals [87]. They adopted amplitude combinations of different channels during straining muscles. The similar method was defined as proportional control [48]. The performance in classifying 10 functions with a linear discriminant classifier, reaching 94%, 93% and 87% at 16, 8 and 4 channels, respectively. In Ishii’s work [49], the combinations of different motions corresponded to eight control commands based on the state machines. 

In our work, Paradigm 3 and 4 were similar with the proportional and state-machine control. Our vehicle could move to five directions with two channels. The travel time of Paradigm 4 was slightly shorter than Paradigm 3. These two paradigms were more sophisticated than Paradigm 2, but they were closer to the real-life control method. However, these paradigms were hard to remember or implement for some subjects, especially for the high-dimensional control.

### 7.4. Limitations and Future Work

There are several basic limitations associated with this study that need further development to provide the wearable sEMG system for clinical purposes. (1) Although we obtained a stable acquisition system, Balouchestni et al. [76] designed a system to recover the original bio-signals with good level of accuracy and SNR greater than 95.8 dB. Therefore, the circuit architecture optimization are still needed. (2) From Table 10, the next step of research should extend the motion pool. (3) The current research study recorded and analyzed the sEMG data performed only by healthy subjects. (4) In the future, minimization of analysis windows and improvement of single-window performance are main works for real-time algorithms. (5) In this study, subjects controlled the designed telecar in a laboratory environment. We are combining our system with a smart wheelchair. To control it in a complicated real scenario, efforts still need to be made.

## 8. Conclusions

Two wearable sEMG acquisition systems are designed and implemented successfully in this work. The PCB-based prototype can capture four-channel sEMG signals simultaneously from different forearm muscles, and the FPC-based system with two channels are utilized for online control. The system could communicate with a laptop wirelessly through Bluetooth. The high SNR of 61.47 dB and 68.91 dB for these systems ensure the signal quality. Temporal and frequency responses indicate that the system can remove noise and are stable during all motions.

The ECU and ECR channels are selected with 97.45% and 96.55% mean classification accuracies across all pairs of motions and subjects. In single-channel analysis, the FD and RFE methods achieve the optimal accuracy with significantly less features than the EC method (*p* = 0.002 and *p* = 0.003 respectively). For the channel ECU, the average accuracy increases to 97.82% with only 14 features. Accuracies above 98% are achieved comparing the REST state with other states. The FD method produces recognition rates in the range of 94.72% to 98.16% comparing among three motions.

Detailed features are selected according to the level of feature separability provided by the EC, FD and RFE methods. According to qualitative and quantitative weighting analysis, these three methods opt for 31, 23 and 33 features, respectively. The feature selection results also prove that time-domain and time-frequency-domain features provide more discriminative information than frequency-domain features. The FD-based feature subset with RF classifier achieves 96.77% accuracy, which is better than other methods and single-type features referred in some references.

Furthermore, to validate the feasibility of proposed methods, we invited same group of subjects to control the designed toy vehicle using four different paradigms. Subjects can accomplish the online task by joysticks with averaged 45.18 s. For the fixed-moving paradigm, the mean travel time is 46.02 s with 94.79% recognition rate. The results of Paradigm 3 and 4 reveal that these paradigms can improve the maneuverability and provide potentials in more sophisticated paths.

Therefore, all mentioned results suggest that our proposed acquisition systems and algorithms can be used in the HCI research. The future work focuses on recording and discerning more motions to realize the accurate implementation of smart wheelchairs.

## Figures and Tables

**Table 1:** List of sEMG features and dimensions.

Feature Name	Abbreviation	Dimension
Mean Absolute Value	MAV	1
Modified Mean Absolute Value 1	MMAV1	1
Modified Mean Absolute Value 2	MMAV2	1
Root Mean Square	RMS	1
Variance	VAR	1
Waveform Length	WL	1
Zero Crossings	ZC	2
Slope Sign Change	SSC	1
Willison Amplitude	WAMP	5
Simple Square Integral	SSI	1
Histogram of sEMG	HEMG	6
Four-order Auto-Regressive Coefficients	AR	4
Median Frequency	MDF	1
Mean Frequency	MNF	1
Short Time Fourier Transform	STFT	1
Average Power of the Wavelet Coefficients	APWC	7
Standard Deviation of the Wavelet Coefficients	SDWC	7

**Table 2:** Definition of muscular movements and corresponding commands to vehicle.

Muscular Movements	Vehicle Motions
Make a Fist (MF)	Move forward by the length of 12 cm
Keep Relaxed (REST)	Move backward by the length of 12 cm
Wrist Extension (WE)	Clockwise rotation of 45 degrees
Wrist Flexion (WF)	Anti-clockwise rotation of 45 degrees
No continuous same results come	Stop

**Table 3:** Definition of muscular movements and control method in Paradigm 3 and 4.

Muscular Movements	Control Method	Vehicle Motions
Make a Fist (MF)	Ch1 = Ch2 = 1	Forward
Keep Relaxed (REST)	Ch1 = Ch2 = 2	Backward
Wrist Extension (WE)	Ch1 = Ch2 = 3	Clockwise rotation
Wrist Flexion (WF)	Ch1 = Ch2 = 4	Anti-clockwise rotation
Others	Ch1 ≠ Ch2	Stop

**Table 4:** Definition of muscular movements and control method in Paradigm 5 and 6.

Muscular Movements	Vehicle Motions
Keep Relaxed (REST)	Switching: on/off
Wrist Flexion (WF)	On: Forward Off: Anti-clockwise rotation
Wrist Extension (WE)	On: Backward Off: Clockwise rotation
Make a Fist (MF)	Stop

**Table 5:** Classification accuracy of each channel for different pairs of movements.

Motion Pairs	APL	ECR	ECU	FCU
REST vs. MF	99.32%	99.42%	99.56%	99.47%
REST vs. WE	96.17%	98.99%	97.98%	99.30%
REST vs. WF	98.86%	99.13%	98.64%	99.34%
MF vs. WE	90.62%	95.16%	93.90%	94.96%
MF vs. WF	84.95%	91.08%	96.86%	91.31%
WE vs. WF	82.32%	95.52%	97.76%	85.62%
Average	92.04%	96.55%	97.45%	95.00%

**Table 6:** Mean optimal feature numbers and classification accuracies of the channel ECU.

Motion Pairs	OFN with EC	MCA with EC	OFN with FD	MCA with FD	OFN with RFE	MCA with RFE
REST vs. MF	19.3	99.73%	3.1	99.73%	1.4	99.73%
REST vs. WE	24.8	98.16%	5.8	98.24%	5.8	98.36%
REST vs. WF	15.2	98.69%	1.8	98.79%	2.4	98.89%
MF vs. WE	28.8	94.52%	26.4	94.72%	23.1	94.68%
MF vs. WF	22.2	97.35%	20.8	97.25%	21.3	97.11%
WE vs. WF	29.9	98.06%	26.1	98.16%	27.8	98.15%
Average	23.4	97.75%	14.0	97.82%	13.6	97.82%

**Table 7:** The selected features and SV rank.

Rank	EC Method		FD Method		RFE Method	
	Feature	Aver. SV	Feature	Aver. SV	Feature	Aver. T_32_
1	APWC_D3	0.2296	WAMP5	1.9005	VAR	18
2	APWC_D2	0.3066	WAMP4	1.7716	WAMP1	18
3	APWC_D4	0.3447	ZC2	1.5245	WAMP3	18
4	APWC_D1	0.3657	ZC1	1.4595	APWC_D1	18
5	STFT	0.3665	WAMP3	1.4290	APWC_D2	18
6	APWC_D5	0.3723	SSC	1.2673	APWC_D3	18
7	VAR	0.3882	SDWC_D1	1.2508	APWC_D4	18
8	SSI	0.3883	SDWC_D3	1.2128	WAMP2	17
9	APWC_D6	0.5859	SDWC_D2	1.2079	SSI	17
10	APWC_A6	0.6015	APWC_D1	1.1998	HEMG4	17
11	WAMP1	0.8886	HEMG1	1.1997	HEMG5	17
12	WAMP2	0.8907	HEMG2	1.1963	STFT	17
13	SDWC_D3	0.9152	MAV	1.1721	APWC_A6	17
14	MMAV2	0.9875	RMS	1.1626	APWC_D5	17
15	SDWC_D4	0.9898	MMAV1	1.1620	APWC_D6	17
16	MMAV1	0.9905	APWC_D3	1.1595	SDWC_D3	17
17	MAV	0.9908	WAMP3	1.1477	MAV	16
18	SDWC_D2	0.9915	APWC_D2	1.1458	MMAV1	16
19	RMS	0.9972	SDWC_D4	1.0873	MMAV2	16
20	SDWC_D5	1.0365	MMAV2	1.0643	RMS	16
21	SDWC_D1	1.0448	WL	1.0626	SDWC_A6	16
22	HEMG4	1.0605	SSI	1.0454	WAMP4	15
23	HEMG5	1.0714	VAR	1.0454	WAMP5	15
24	SDWC_A6	1.2020			SDWC_D1	15
25	SDWC_D6	1.2354			SDWC_D2	15
26	WAMP3	1.2441			SDWC_D4	15
27	WL	1.2672			SDWC_D6	15
28	SSC	1.3096			ZC2	14
29	HEMG3	1.4682			SDWC_D5	14
30	ZC2	1.4725			HEMG3	13
31	HEMG6	1.5413			HEMG6	13
32					HEMG1	11
33					HEMG2	11

**Table 8:** Classification performance of machine learning algorithms. ACC is the abbreviation of Accuracy.

Features	kNN		ANN		RF		SVM	
	ACC	F-Score	ACC	F-Score	ACC	F-Score	ACC	F-Score
EC	94.73%	0.9473	**96.74%**	0.9672	96.66%	0.9656	95.37%	0.9533
FD	94.41%	0.9441	96.67%	0.9667	**96.77%**	0.9669	95.40%	0.9529
RFE	94.37%	0.9436	**95.82%**	0.9678	95.60%	0.9651	95.01%	0.9489
RMS	86.36%	0.8630	87.75%	0.8723	**89.43%**	0.8918	89.14%	0.8755
MAV	87.23%	0.8719	87.21%	0.8664	**89.12%**	0.8885	88.20%	0.8773
APWC	86.08%	0.8585	90.20%	0.9007	**91.63%**	0.9150	87.07%	0.8678
SDWC	85.36%	0.8535	88.90%	0.8861	**91.60%**	0.9150	82.85%	0.8004

**Table 9:** The recognition rate and travel time for different online paradigms.

Subject	Paradigm 1		Paradigm 2		Paradigm 3		Paradigm 4	
	TT (s)	RR	TT (s)	RR	TT (s)	RR	TT (s)	RR
S1	44.98	--	44.88	99.25%	47.21	96.88%	46.93	--
S2	45.86	--	44.82	98.38%	50.31	89.61%	46.42	--
S3	44.44	--	44.94	97.66%	45.07	98.03%	46.29	--
S4	45.08	--	46.93	95.75%	48.87	95.77%	47.08	--
S5	44.73	--	46.60	96.00%	47.10	92.50%	49.61	--
S6	45.55	--	46.86	92.19%	51.43	84.45%	47.81	--
S7	44.82	--	45.52	95.91%	48.87	96.09%	46.97	--
S8	45.70	--	47.04	86.16%	52.03	85.08%	51.37	--
S9	45.46	--	46.59	91.77%	50.67	88.54%	50.26	--
Mean	45.18	--	46.02	94.79%	49.06	91.77%	48.08	--

**Table 10:** Comparison of results and methods with other studies using sEMG-based signals.

Year	Features	Feature Selction Classification	ACC (%)	Applications	Classes	Channels
2015 [12]	DWT	RF	96.67	Diagnostic Decision	3	5
2017 [26]	MAV	SVM	>90	Prosthetic Hands	4	1
2017 [29]	RMS & WL	LDA	90%	Prosthetic Hands	6	4
2015 [32]	RMS & DRMS	Fuzzy C-Means	89.15	Motions Recognition	4	16
2012 [35]	AR & DWT	ANFIS ^1^	95	Diagnostic Decision	3	1
2014 [36]	MUSIC ^2^	SVM	92.55	Diagnostic Decision	3	5
2017 [37]	WPD	RF	92.1	Motions Recognition	10	8
2017 [40]	178	Fisher Criterion + SVM	84.01	Gait Phase Recognition	4	4
2017 [42]	21 TD ^3^ & 6 FD ^4^	RFE + Extreme Learning Machine	>90	Motions Recognition	17	12
2017 [43]	15	SVM	63–99	Prosthetic Hands	6	1
2017 [44]	MAV	ANN	94	Virtual Trackpad	10	4
2017 [69]	DFT & MAV	SVM	70.2	Motions Recognition	14	8
2017 [81]	22	RES ^5^ + LBN ^6^	93.25	Speech Recognition	11	5
This work	46	FD + RF	96.77	Motions Recognition	4	2

^1^ ANFIS: the Adaptive Neuro-Fuzzy Inference System. ^2^ MUSIC: feature extraction using Multiple Signal Classification. ^3^ TD: Time Domain. ^4^ FD: Frequency Domain. ^5^ RES: ratio of Euclidian distance and standard deviation. ^6^ LBN: Linear Bayes Normal classifier.
